# Performance of Non-invasive Biomarkers of Liver Fibrosis Amongst Hispanics and African Americans

**DOI:** 10.7759/cureus.35032

**Published:** 2023-02-15

**Authors:** Kesiena Akpoigbe, Khushbir Bath, Alvaro Genao, Joan Culpepper-Morgan

**Affiliations:** 1 Internal Medicine, Harlem Hospital Center, Columbia University College of Physicians and Surgeons, New York City, USA; 2 Gastroenterology, Harlem Hospital Center, Columbia University College of Physicians and Surgeons, New York City, USA

**Keywords:** liver cirrhosis, liver fibrosis, liver biopsy, minority population, receiver operating curve, biomarkers of liver fibrosis

## Abstract

Introduction: Liver biopsy is the gold standard for fibrosis staging. However, it is limited by significant complications. Non-invasive markers of fibrosis have been developed as an alternative to liver biopsy. The performance of these different markers varies with the etiology of liver fibrosis and possibly amongst different ethnicities. We aim to assess the performance of non-invasive markers of liver fibrosis amongst Hispanics and African Americans.

Method: This is a retrospective cohort analysis of patients who had liver biopsy as part of their evaluation of chronic liver disease. One hundred and twenty-six records were analyzed. Univariate and multivariate analyses were performed. Probit regression receiver operating characteristic curve analysis was used to assess the sensitivity of the different non-invasive biomarkers and underlying variables with respect to liver biopsy. The following non-invasive markers for fibrosis were used: Fibrosis-4 (FIB-4), aspartate aminotransferase (AST) to platelet ratio index (APRI), age-platelet, AST/alanine aminotransferase (AST/ALT) ratio, fibrosis cirrhosis index (FCI), and fibrosis index (FI).

Results: About two-thirds of the study population were African Americans with majority of the study population having chronic liver disease from viral infection. Minimal to no fibrosis by the METAVIR (an acronym for Meta-analysis of Histological Data in Viral Hepatitis) score was found in 58% of patients compared to 42% with moderate to severe fibrosis. Hispanics were more likely than Blacks to have hepatic steatosis. Age significantly increased the sensitivity and specificity of APRI and age-platelet scores. The AST/ALT score had a lower sensitivity for liver fibrosis in women compared to men in our study population. The sensitivity of FIB-4 and age-platelet was higher in Hispanics compared to African Americans while the opposite was the case for APRI, AST/ALT, FCI, and FI.

Conclusion: Non-invasive biomarkers are useful in detecting liver fibrosis. FIB-4 and age-platelet have a high sensitivity in Hispanics while African Americans have a high sensitivity for APRI, AST/ALT, FCI, and FI. It is worth noting that these non-invasive biomarkers had variable performances when ethnicity, age, and sex were considered in our population.

## Introduction

Liver biopsy is the gold standard for fibrosis staging despite being an invasive procedure and subject to sample variability and a significant incidence of complications [1-8]. Recently, its effectiveness has been challenged by non-invasive markers of liver fibrosis with their ability to predict the extent of fibrosis and exclude the need for liver biopsy. These markers are surrogates for liver inflammation and function and utilize easily available biochemical information when used in combination.

Scores developed from these biomarkers include Fibrosis-4 (FIB-4), aspartate aminotransferase (AST) to platelet ratio index (APRI), age-platelet, AST/alanine aminotransferase (AST/ALT) ratio, fibrosis cirrhosis index (FCI), and fibrosis index (FI). In addition, the fibrosis score accuracy may vary with the etiology of liver disease [9-14]. Amongst the different biomarkers, their performances varies in the prediction of liver fibrosis [15-18]. AST/ALT and FIB-4 are better predictors of non-alcoholic fatty liver disease (NAFLD), and FCI is a good predictor of cirrhosis in chronic hepatitis C infection than APRI, FIB-4, and FI.

It is known that Hispanics have a high rate of NAFLD and liver cirrhosis than Caucasian/African Americans. Conversely, African Americans have the lowest reported rate of NALFD/liver fibrosis. It is possible that the difference in these rates in Hispanics may be due to patatin-like phospholipase domain containing 3 (PNPLA3) gene expression in this population [19]. Harlem Hospital Center (New York City) serves a population that is 60% Black and 30% Hispanic, and 10% from other ethnicities. Our aim was to determine if these non-invasive markers of liver fibrosis performed differently in these two populations representing opposite ends of the NAFLD spectrum.

## Materials and methods

Study design and patients

This was a single-center, retrospective cohort analysis of Harlem Hospital patients who had had liver biopsy as part of their evaluation of chronic liver disease between July 1, 2008, and December 31, 2014. One hundred and forty-five biopsies were performed. Nineteen samples were excluded because of insufficient tissue or fragmentation of the biopsy specimen. Deidentified data for the remaining 126 was obtained from the electronic medical record system. Demographic variables were recorded for each patient. These included age at diagnosis, sex, race, and ethnicity.

Liver biopsy specimens and staging of fibrosis

Liver specimens were staged based on the METAVIR (an acronym for Meta-analysis of Histological Data in Viral Hepatitis) score, and were further dichotomized into nil to minimal fibrosis and moderate to severe fibrosis. Those with nil to minimal fibrosis (F0-1) had scores of 0-1 while those with moderate to severe fibrosis (F2-4) had scores of 2-4.

Non-invasive biomarkers of fibrosis

Supplementary data collected within six months of liver biopsy was used to calculate the scores (Table 1).

**Table 1:** Non-invasive biomarkers of liver fibrosis FIB-4, Fibrosis-4; APRI, aspartate aminotransferase to platelet ratio index; AST/ALT, aspartate aminotransferase/alanine aminotransferase; FCI, fibrosis cirrhosis index; FI, fibrosis index; PLT, platelet; ULN, upper limit of normal

Non-invasive biomarkers	Calculation of scores
FIB-4	(Age (years) x AST (U/L))/(Platelet count (10^9^/L) x ALT (10^9^)^1/2^) [20]
APRI	(AST (U/L)/ULN of AST) x 100/Platelet count (10^9^/L) [21]
FCI	(ALP x Bilirubin)/(Albumin x Platelet count) [22]
FI	8.0 – 0.01 x PLT (10^9^/L) – Serum albumin (g/dL) [22]
AST/ALT	AST/ALT [22]
Age-platelet	Age/Platelet [23]

Statistical analysis

Baseline demographic information analysis was contingent on the type of data. Means and standard deviations were calculated for continuous data, and in some cases the 95% confidence interval (CI) was inferred. For categorical variables, percentages and in some instances, proportions were obtained. Mean scores of the non-invasive biomarkers with respect to the METAVIR stage were calculated. A chi-square test of independence was performed to examine the relationship between race and the underlying liver pathology. Unpaired t-tests were done to show the difference between mean scores. We used probit receiver operating characteristic (ROC) curve regression to model the sensitivity and specificity of the individual non-invasive biomarkers in predicting liver fibrosis, adjusting for age, sex, race, and the underlying liver pathology. The area under the ROC curve (AUROC) of the different biomarkers was estimated and compared to the liver biopsy to assess their sensitivity and specificity. The Liu method was used to estimate a feasible cut-off point for different biomarkers and their sensitivity, specificity, and likelihood ratio [24]. All statistical tests were considered significant at p<0.05. Analysis was conducted using Stata statistical software, version 13 (StataCorp, TX).

## Results

Patient demographics

Baseline characteristics of the study population are given in Table 2.

**Table 2:** Demographic characteristics of the study population ^a^Includes HIV, and hepatitis C and B

Characteristics	Total (n=126)
Age, mean (SD)	49.7 ± 11.0
Sex, n (%)	
Male	81 (64.3%)
Female	45 (35.7%)
Race, n (%)	
African American	92 (73.0%)
Hispanic	34 (27.0%)
Fibrosis n (%)	
Nil to minimal fibrosis (Grade 0-1)	73 (57.9%)
Moderate to severe fibrosis (Grade 2-4)	53 (43.1%)
Co-morbidity, n (%)	
Viral infection^a^	91 (72.2%)
Hepatic steatosis	35 (27.8%)

The mean age was 49.7 years with the majority being male (64.3%). Blacks comprised 73% of the study population and Hispanics 27%. The majority of the study population had some viral infections, either HIV infection and/or hepatitis B or C. Minimal to no fibrosis and moderate to severe fibrosis as determined by the METAVIR score were seen in 58% and 42%, respectively, of the study population.

Relationship between race/ethnicity, liver pathology, and fibrosis

The relationship between race and the underlying liver pathology was significant, χ^2 ^(1, N=126) = 11.4, p=0.001. Hispanics were more likely than Blacks to have hepatic steatosis (Table 3).

**Table 3:** Association between race and liver pathology n, number of cases; df, degree of freedom ^a^Chi-square test of independence

Race	Viral infection n (%)	Steatosis n (%)	n	χ^2 ^statistic^a^ (df)	p-value^a^
African American	74 (80.4)	18 (19.7)	92	11.5 (1)	0.01
Hispanic	17 (50.0)	17 (50.0)	34		

**Figure 1:**
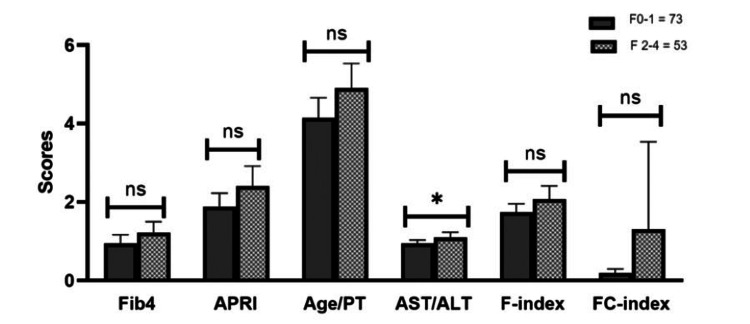
Association between non-invasive biomarkers and liver fibrosis Fib4, Fibrosis-4; APRI, aspartate aminotransferase to platelet ratio index; age/PT, age-platelet; AST/ALT, aspartate aminotransferase/alanine aminotransferase; FC-index, fibrosis cirrhosis index; F-index, fibrosis index; ns, not  significant F0-1: nil to minimal fibrosis F2-4: moderate to severe fibrosis *p<0.05

When all etiologies were pooled, the mean difference of 0.14 in scores between F2-4 and F1-0 fibrosis for AST/ALT was marginally significant (p=0.05). The other non-invasive biomarker scores including FIB-4, APRI, age-platelet, FI, and FCI were not significant, as shown in Figure 1.

Predictors of sensitivity and specificity of non-invasive biomarkers of liver fibrosis

Age was an influencing factor in the sensitivity and specificity of non-invasive biomarkers of liver fibrosis. Those aged 65 year or older had a significantly increased sensitivity of APRI (adjusted coefficient, or aCoef, 2.69, CI 0.14-5.23, p=0.04) and age-platelet score (aCoef 5.07, CI 2.22-7.92, p=0.00). Those aged 36-64 years showed a significantly increased sensitivity of age-platelet score (aCoef 3.22, CI 1.28-5.18, p=0.001) when compared to those less than 35 years of age (Table 3). Females were found to have a significantly decreased sensitivity of AST/ALT when compared to males (Table 4). The sensitivity of FIB-4 and age-platelet was higher in Hispanics compared to African Americans and the sensitivity of APRI, AST/ALT, FCI, and FI was decreased in Hispanics versus African Americans (Tables 4, 5).

**Table 4:** ROC curve regression adjusting for sensitivity and specificity of non-invasive biomarkers amongst predictors AST/ALT, aspartate aminotransferase/alanine aminotransferase; CI, confidence interval; ROC, receiver operating characteristic ^†^Adjusted coefficient significant for p<0.05

	n (%)		AST/ALT		Fibrosis index		Fibrosis cirrhosis index	
	126 (100)		aCoef^†^ (95% CI)	p	aCoef^† ^(95% CI)	p	aCoef^†^ (95% CI)	p
Age (years)								
≤35		14 (11.1)	Reference		Reference		Reference	
36-64	102 (81.0)		0.02 (-0.46-0.51)	0.92	0.67 (-0.48-1.83)	0.25	1.45 (-6.48-9.39)	0.72
≥65	10 (8.0)		0.01 (-0.70-0.73)	0.97	0.31 (-1.37-2.00)	0.71	0.07 (-11.52-11.67)	0.99
Sex								
Male	81 (64.3)		Reference		Reference		Reference	
Female	45 (35.7)		-0.14 (-0.27--0.00)	0.049	-0.14 (-0.52-0.24)	0.48	-0.04 (-0.25-0.16)	0.67
Race								
African American		92 (73.0)	Reference		Reference		Reference	
Hispanic	34 (27.0)		-0.29 (-0.41-0.03)	0.00	-0.05 (-0.47-0.36)	0.80	-0.23 (-0.25-0.21)	0.84
Co-morbidity								
Virus	91 (72.2)		Reference		Reference		Reference	
Steatosis	35 (27.8)		0.03 (-0.12-0.19)	0.67	0.09 (-0.35-0.53)	0.69	0.08 (-0.14--0.30)	0.46

**Table 5:** ROC curve regression adjusting for sensitivity and specificity of non-invasive biomarkers amongst predictors FIB-4, Fibrosis-4; APRI, aspartate aminotransferase to platelet ratio index; ROC, receiver operating characteristic; CI, confidence interval ^†^Adjusted coefficient significant for p<0.05

	n (%)	FIB-4		APRI		Age-platelet index	
	126 (100)	aCoef^† ^(95% CI)	p	aCoef^†^ (95% CI)	p	aCoef^† ^(95% CI)	p
Age (years)							
≤35	14 (11.1)	Reference		Reference		Reference	
36-64	102 (81.0)	0.74 (-0.27-1.75)	0.15	1.67 (-0.72-3.42)	0.06	3.22 (1.28-5.18)	0.001
≥65	10 (8.0)	0.44 (-1.03-1.91)	0.55	2.69 (0.14-5.23)	0.04	5.07 (2.22-7.92)	0.000
Sex							
Male	81 (64.3)	Reference		Reference		Reference	
Female	45 (35.7)	-1.23 (-0.48-0.23)	0.48	-0.22 (-0.80-0.35)	0.45	-0.27 (-1.06-0.51)	0.49
Race							
African American	92 (73.0)	Reference		Reference		Reference	
Hispanic	34 (27.0)	0.22 (-1.79-0.62)	0.28	-0.13 (-0.77-0.51)	0.69	0.64 (-0.23-1.49)	0.15
Co-morbidity							
Virus	91 (72.2)	Reference		Reference		Reference	
Steatosis	35 (27.8)	-0.29 (-0.69-0.12)	0.41	0.07 (-0.60-0.74)	0.84	-0.21 (-1.13-0.72)	0.66

Measuring test accuracy of non-invasive biomarkers of fibrosis

**Figure 2:**
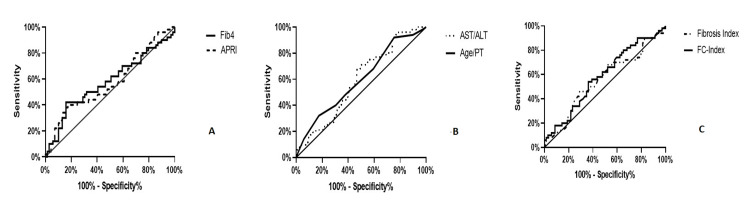
ROC curves of non-invasive biomarkers ROC curves for (A) Fib4 and APRI; (B) age/PT and AST/ALT; and (C) FC-index and fibrosis index ROC, receiver operating characteristic; Fib4, Fibrosis-4; APRI, aspartate aminotransferase to platelet ratio index; AST/ALT, aspartate aminotransferase/alanine aminotransferase; FC-index, fibrosis cirrhosis index; PT, platelet

The AUROC showed FCI (44%) to have a significantly lower sensitivity when compared with FIB-4 (58%), APRI (57%), AST/ALT (61%), age-platelet (60%), and FI (58%) in detecting liver fibrosis for all diagnoses (Figure 2).

AST/ALT and age-platelet appear to be the most sensitive non-invasive biomarkers for detecting liver fibrosis in this study population when compared with FIB-4, APRI, and FI.

Determining a new cut-off point for non-invasive biomarkers of fibrosis

While the study looked at the sensitivity and specificity of different non-invasive biomarkers in estimating liver fibrosis, we also attempted to create a feasible cut-off point to increase the sensitivity for the diagnosis of liver fibrosis. Using the Liu estimate, the maximum cut-off point generated for the biomarkers had a lower sensitivity but a relatively high specificity for FIB-4, age-platelet, and APRI as shown in Table 6.

**Table 6:** Estimating a feasible cut-off point for liver fibrosis diagnosis using non-invasive markers FIB-4, Fibrosis-4; APRI, aspartate aminotransferase to platelet ratio index; ROC, receiver operating characteristic; AST/ALT, aspartate aminotransferase/alanine aminotransferase; AUROC, area under the ROC curve; CI, confidence interval; +LR, positive likelihood ratio; -LR, negative likelihood ratio

	Cut-off point	95% CI	p-value	Sensitivity	Specificity	AUROC	+LR	-LR
FIB-4	1.19	0.79-1.59	0.000	0.42	0.84	0.63	2.63	0.69
APRI	2.44	1.56-3.32	0.000	0.40	0.83	0.61	2.35	0.72
Age-platelet	4.00	2.04-5.96	0.000	0.48	0.62	0.55	1.26	0.84
AST/ALT	0.87	0.83-0.91	0.000	0.71	0.51	0.61	1.45	0.57
Fibrosis index	1.93	1.53-2.33	0.000	0.54	0.64	0.59	1.50	0.72
Fibrosis cirrhosis index	0.12	0.04-0.19	0.001	0.28	0.77	0.52	1.22	0.94

AST/ALT had the highest sensitivity but a moderate specificity. They all had in common low positive and negative likelihood ratio values of approximately 15% except for FCI and age-platelet scores with a higher negative likelihood ratio (45%).

## Discussion

Saab et al. suggested that the difference in the prevalence of fatty liver disease between African Americans and Hispanic Americans is related to the difference in the prevalence of the I148M single nucleotide polymorphism of the PNPLA3 gene [19]. This is a protein involved in lipid metabolism. The allelic frequency is 0.49 for Hispanics, 0.23 for European Americans, and 0.17 for African Americans. It is notable that the prevalence of nonalcoholic steatohepatitis varies within the Hispanic population, being the highest in Mexicans (33%) and Central American Hispanics (25%) and lowest in Caribbean (Peurto Rico, Dominican Republic, Cuba) Hispanics (16%-18%). Our population consisted mostly of Caribbean Hispanics. Twenty-seven percent of our cohort were Hispanics. Steatosis was present in 50% of the Hispanic patients but only 20% of the African American patients confirming the finding of Saab et al.

Contrary to the meta-analysis by Shaheen and Myers, we used a high recommended cut-off of 2.44 for APRI (CI 1.56-3.32) [9]. This improved the specificity to 83% at the expense of sensitivity decreasing to 40% in our total cohort, similar to the meta-analysis by Lin et al. who used a cut-off of 2.00 for APRI but they calculated a specificity of 91% with a sensitivity of 46% [14]. Thus, APRI underperformed in our cohort of mixed diagnosis. One study noted the underperformance of APRI in late-stage HIV-AIDS due to AIDS-induced thrombocytopenia [12]. However, HIV infection was found in only 6% cases of our cohort.

FCI is reported to be a better predictor of liver cirrhosis in viral infection (chronic hepatitis C) than APRI, FIB-4, and FI [11]. This was not the case in our study. In those with viral infection including hepatitis B and C, FIB-4 and age-platelet were more sensitive for the diagnosis of liver fibrosis. However, it should be noted that the FCI study by Ahmad et al. was strictly on chronic hepatitis C. Our study categorized all viral infections including hepatitis B, C, and HIV into a single category and these could have had a confounding effect on the outcome as compared to the FCI study.

To increase the sensitivity and specificity of these tests, we created new cut-offs and assessed how the different non-invasive biomarkers performed. The sensitivity and specificity based on the new cut-off of FIB-4 (1.19), APRI (2.44), age-platelet (4.00), AST/ALT (0.87), FI (1.93), and FCI (0.12) were all increased as shown by their respective AUROCs and likelihood ratio tests. This increase in cut-offs was associated with an increase in the specificity for the individual biomarkers but a decrease in sensitivity. However, their positive likelihood ratios were significantly increased. Of note was that the increase in the AUROC for FIB-4 (63%), APRI (61%), AST/ALT (61%), and FI (59%) was not high enough to improve their overall sensitivity in diagnosing fibrosis in this minority community.

There were also limitations present in the study. First, given the small sample size of the study, it was difficult to draw inferences when differences were noted but were not statistically significant, and therefore, we might have underestimated the differences in the performance of these tests in this minority population. Also, the grouping of viral infections, which include hepatitis B, C, and HIV, could have confounded the effect the individual virus would have had on the different non-invasive biomarkers.

## Conclusions

In conclusion, these biomarkers may be more useful in detecting liver fibrosis; however, it is unclear if the non-invasive blood-test-based fibrosis measures could replace the gold standard liver biopsy, given the discrepancy in the findings amongst our patient cohort. A future comparison of non-invasive biomarkers with elastography, which is also a non-invasive test, in these groups would be useful; it could also be another way to determine other tests that could be employed. In addition, it could also account for changes that did not reach statistical significance in this study. More larger studies will be needed to confirm these findings.
